# Telomere-to-telomere genome assembly of Microsporidia sp. MB, a microsporidian symbiont of *Anopheles coluzzii* isolated from Burkina Faso

**DOI:** 10.1186/s12864-026-12799-4

**Published:** 2026-04-09

**Authors:** Roland Pevsner, Julien Martinez, Deepak-Kumar Purusothaman, Beth C. Poulton, Abdelhakeem I. Adam, Ewan R.S. Parry, Saré Issiaka, Abdoulaye Diabaté, Steven P. Sinkins

**Affiliations:** 1 MRC Centre for Virus Research, University of Glasgow, Glasgow, UK; 2 Institut de Recherche en Science de la Santé, Bobo-Dioulasso, Burkina Faso; 3 Present Address: Tree of Life, Wellcome Sanger Institute, Hinxton, England, UK; 4 Present Address: School of Biological and Medical Sciences, Oxford Brookes University, Oxford, UK

**Keywords:** Microsporidia, Microsporidia sp. MB, Illumina, PacBio, Genome sequencing, CpG methylation, Tetraploidy, Telomere

## Abstract

**Background:**

Microsporidia sp. MB is an intracellular parasite of anopheline mosquitoes identified across the African continent. Microsporidia sp. MB infections appear to have no significant effect on mosquito fitness and can vertically transmit, whilst infected individuals have exhibited significantly reduced levels of *Plasmodium falciparum* transmission. These combined characteristics make Microsporidia sp. MB a promising candidate for use in malaria control. A comprehensive genome would greatly facilitate investigation into the evolution and biological pathways underlying important phenotypes, such as the mechanism of malarial inhibition.

**Results:**

In this study, we present the *de novo* assembly of the first complete genome of Microsporidia sp. MB SOUVK7. Multi-platform sequencing was performed on ovary samples of laboratory established *Anopheles coluzzii* collected from Burkina Faso. The SOUVK7 genome has a total size of 9.16 Mb, encodes 2,435 genes and is organised into 13 chromosomes with telomeres identified at all flanks. Telomeric repeats exhibit a 4-mer motif due to a glutamine deletion previously unobserved in Microsporidia. Ploidy analysis of Illumina reads predicts Microsporidia sp. MB as tetraploid, whilst analysis of CpG methylation and retroelements highlights loci in all chromosomes with characteristics consistent with regional centromeres. Orthology analysis identifies several key genes in pathways associated to telomeric and centromeric maintenance, along with methylation and host invasion machinery. A loss of several components of the infection machinery is observed in Microsporidia sp. MB and the wider Enterocytozoonida, consistent with a general trend towards genome size reduction in the clade.

**Conclusion:**

This study provides the first complete, telomere to telomere assembly of Microsporidia sp. MB, offering new insight into the genomic architecture of Microsporidia sp. MB and the broader Mrazekiidae family.

**Supplementary Information:**

The online version contains supplementary material available at 10.1186/s12864-026-12799-4.

## Background

Pathogenic species of the Microsporidia are emerging concerns in public health, sericulture, and aquaculture sectors [1–3]. This phylum of obligate intracellular parasites infect a broad range of hosts across all animals, exhibiting a variety of symptoms from digestive distress to growth retardation and high mortality [4–6]. Microsporidia are generally observed to be opportunistic pathogens, microorganisms where infection and pathogenesis often present in hosts with low or compromised immunity. This is exemplified by many notable microsporidian diseases being observed in high-intensity animal agriculture and immuno-compromised patients [2, 7, 8]. Due to the opportunistic pathology of Microsporidia, very few symbiotic relationships have been reported with their hosts. Microsporidia sp. MB (MB) was recently discovered in several species of *Anopheles* mosquitoes in Kenya and has also been reported in mosquito field populations across sub-Saharan Africa [9–12]. In Kenya, mosquitoes infected with MB and challenged with malaria-infected patient blood had reduced rates of *Plasmodium* infection in their midgut and salivary glands when compared to MB-negative mosquitoes [10]. This data was also corroborated by a lack of MB/*Plasmodium* coinfections being reported in extensive field sampling of wild *Anopheles* mosquitoes. These results strongly indicate a malarial inhibition effect with *Anopheles* mosquitoes, provided by MB. As MB appears to confer no significant costs to mosquito host longevity or fecundity, transmits maternally, and exhibits a symbiotic or commensal relationship, MB shows great potential as an alternative agent in malaria control.

The Microsporidia phylum is part of the Opisthosporidia, a superphylum also including the Rozellomycota (Cryptomycota) and Aphelidea phyla, which is often considered a sister clade of Fungi [13]. The boundaries between Microsporidia species and those of the neighbouring Rozellamycota remain in debate due to shared morphological and metabolic characteristics, with general consensus considering approximate limits of the phylum at the *Metchnikovella*,* Mitosporidium* and *Rozella* genera [13–15]. Amongst the ‘canonical’ or ‘short-branch’ Microsporidia, taxonomy is described by taxonomic families and a broader taxonomical framework of clades [14]. Initial small subunit ribosomal RNA sequencing of MB reported a phylogeny within the Mrazekiidae family (Enterocytozoonida clade), with closest relative species of *Crispospora chironomi* and *Vittaforma corneae* [10]. The Mrazekiidae family includes several other species capable of infecting insects and other invertebrates, including midges (*C. chironomi*) [16], moths (*Endoreticulatus bombycis)* [17], and planktonic crustaceans (*Glugoides intestinalis*) [18]. The broader Enterocytozoonida clade also includes the Enterocytozoonidae family, which compromised a species infecting marine invertebrates [5, 7, 19].

Microsporidia exhibit extensive levels of genomic reduction of both coding and non-coding regions, believed to be the result of their obligate parasitic lifestyles [20]. For this reason, Microsporidia species still have the smallest reported complete genomes of any eukaryotic organism, as seen in *Encephalitozoon intestinalis* (2.61 Mb) [21]. The reduction in total genome size is also reflected in low total gene counts of approximately 1,200 to 6,000 (*Dictyocoela roeselum*, GenBank: ASM1625598v1; *Dictyocoela muelleri*, GenBank: ASM1625607v1, respectively). Phylum-wide analysis has previously suggested that approximately 48% of gene families within Microsporidia are unique to their phylum and are often even specific to families or clades [20]. As observed in other intracellular parasites, Microsporidia genomes typically exhibit low GC contents for eukaryotic organisms with mean GC% of available Microsporidia genomes (S4 Table) being only 34.5%, thus often making them easy to differentiate from the reads of their metazoan host [18, 22, 23]. Currently our understanding of microsporidian chromosomal architecture is limited due to the low number of published complete or chromosome level assemblies (NCBI: 1 complete and 6 chromosome-level assemblies respectively; as of Feb 2026). Assembly sizes vary between 2.2 and 21.6 Mb (*Encephalitozoon romaleae*, GenBank: GCA_000280035.2; *Hamiltosporidium tvaerminnensis*, GenBank : GCA_022605425.2, respectively), however the only complete telomere-to-telomere assembly (*Vairimorpha necatrix*, GenBank: GCA_036630325.1) is reported at 15.0 Mb in size. Similarly, scaffold numbers within chromosome-level genomes are seen to vary between 11 and 18 ( *E. intestinalis*, GenBank GCA_000146465.1; *Antonospora locustae*, GenBank: GCA_007674295.1, respectively), with the assembly of *V. necatrix* confirming 12 full chromosomes. As research initiatives continue to assess MB’s potential as an agent for malaria control, a comprehensive and complete genome is needed to support investigation into the mechanisms of *Plasmodium* inhibition and host interaction, along with population genetics and diversity across tropical Africa. Four MB genome assemblies have previously been released, with the most complete of which being from the AHL03 strain of MB, but all assemblies are highly fragmented (≤ 2,400 contigs) and thus incompatible for analysis of genome synteny or chromosomal architecture [24]. These genomes are all assembled from separate collections in northern Kenya, and each likely represent populations with low genetic differentiation to one another, complicating efforts to identify effective genetic markers for population analysis.

In this article, we present the first gap-less, telomere-to-telomere *de novo* assembly of the Microsporidia sp. MB genome, which was isolated from laboratory established *Anopheles coluzzii* collected in Burkina Faso. This expands the diversity of available MB genomes but also acts as a new reference for investigation of microsporidian genome structure and architecture, and can aid reassembly of prior MB genome releases. Furthermore, given the low number of chromosome level and complete genomes within the Microsporidia phylum, this novel assembly expands our genomic understanding of the wider Enterocytozoonida clade [14].

## Materials and methods

### Mosquito rearing and material isolation

Mosquito tissues colonised by Microsporidia sp. MB were isolated from a previously lab-adapted *Anopheles coluzzii* colony, originating from Burkina Faso. Rearing and sampling for this study was performed at the MRC-University of Glasgow Centre for Virus Research, University of Glasgow, Glasgow, UK. Mosquitoes were maintained at 28 °C at a constant 80% relative humidity with 12-hour day/night light cycles. Early instar larvae were fed on liver powder (Now Foods, Bloomington, IL, USA) and progressed to TabiMin tablets (Tetra, Melle, Germany) from second instar. Adults were reared in cages of 500–2000 individuals and provided 5% sucrose solution *ad libitum*. Four days post-eclosure, adult females were fed with human blood (Scottish National Blood Transfusion Service, Glasgow, UK) administered through a membrane feeder system (Hemotek ltd, Blackburn, UK) and males and non-blood fed females were removed. Moist filter paper cones were placed into darkened cages 96 h post-bloodmeal to promote immediate egg laying behaviour.

### DNA isolation and sample verification

PacBio sequencing was performed on dissected ovaries from gravid females, 96 h post-bloodmeal. In an attempt to maximise the ratio of MB reads to host background reads, gravid ovary tissues and recently laid eggs were previously identified to have the highest density of meronts and sporonts with the tissues and so, these tissues were selected DNA extraction [25]. Ovarian tissues were selected specifically for PacBio due to it’s higher yields of high molecular weight (HMW; >50 kb) DNA, compared to early egg extractions. Females were anaesthetised through cold exposure and gravid ovaries were dissected individually in phosphate-buffered saline (PBS) solution, with PBS being changed between dissections. Ovaries from 100 females were pooled and transferred to − 20 °C for storage. DNA extraction was performed on 200 ovaries, using the Gentra Puregene Tissue kit (4 g; QIAGEN N.V., Hilden, Germany) following the provided ‘DNA Purification from Tissue’ protocol in order to purify HMW DNA only. A final sample of 10 µg total HMW DNA mass was targeted, as prior sequencing of MB suggested very low proportions of MB to host reads (~ 1.2% of total reads) [24, 26]. The HMW DNA sample was eluted into 50 µl of molecular-grade water and stored at 4 °C until submission to Edinburgh Genomics, University of Edinburgh, UK.

As previously discussed, Illumina sequencing for ploidy analysis was performed on sterilised, early-stage eggs to maximise the MB-host DNA ratio [25]. As HMW DNA was unnecessary for Illumina sequencing, recently laid eggs could be scaled for greater quantities of material and could be sterilised to minimise bacterial contaminants. To ensure the minimal age of the embryos, egg cones were swapped within gravid female cages hourly for six consecutive hours. Collected eggs were covered and allowed to melanise at room temperature (18–21 °C) for one hour, before being inactivated at − 20 °C for a minimum of one hour. All inactivated egg cones were pooled and stored at − 20 °C until sufficient material was collected. Once approximately 300–500 eggs were collected, all egg cones were thawed, and eggs were washed onto a vacuum membrane filter. The eggs were sterilised for five minutes by submersion in 1% hypochlorite solution (1:10 10% hypochlorite in water), drained through vacuum filtration and continuously washed in sterile PBS for an additional 5 min. Eggs were transferred to a 1.5 ml centrifuge tube and DNA was extracted through a modified Phenol: Chloroform extraction method. Briefly, all liquid was aspirated from the egg suspension and 500 µl of phenol was added to the centrifuge tube. All eggs were ruptured through manual grinding with a micropestle, before adding 500 µl of chloroform and mixing through inversion. The extraction mix was centrifuged (13,000 x g for 10 min at 4 °C), the aqueous layer was collected and the DNA was precipitated with an equal volume of precipitation solution (40 µl 3 M sodium acetate in 800 µl absolute ethanol) for 60 min at − 20 °C. Precipitated DNA was collected through centrifugation (13,000 x g for 10 min at 4 °C), washed in 70% ethanol, eluted into 100 µl molecular-grade water and stored at 4 °C until submission to Glasgow Polyomics (now MVLS Shared Research Facilities), University of Glasgow, UK.

Nanopore sequencing provided additional long-read sequence and CpG methylation data for post-assembly analysis. As with the PacBio sample, 200 gravid ovaries were dissected, pooled in PBS and stored at − 20 °C prior to extraction. Sample DNA was extracted with the Blood & Cell Culture DNA Kit (QIAGEN N.V., Hilden, Germany) following the provided Genomic DNA Preparation methods for 20/G columns. A final sample of 1 µg total HMW DNA mass was targeted, as per recommendations for > 10 kb reads for PromethION flow cells (Oxford Nanopore Technologies, Oxford, UK) [27]. Extracted HMW sample was eluted into 50 µl of molecular-grade water and stored at 4 °C until submission to Edinburgh Genomics, University of Edinburgh, UK.

### Genome sequencing and assembly

A single HMW DNA sample was submitted for PacBio sequencing, with sample quality and average DNA fragment size being assessed through TapeStation (Agilent Technologies ltd), Femto Pulse (Agilent Technologies ltd) and dsDNA QuBit (ThermoFisher Scientific Inc) systems. The sample library was prepared with 10.95 µg of HMW gDNA and sequenced with a Revio SMRTbell prep kit on a Revio SMRT 25 M ZMW cell using HiFi mode (PacBio). Initial read quality and distributions were verified with FastQC [28]. Host contamination was removed by mapping the PacBio HiFi reads to an *An. gambiae* reference genome (NCBI accession: GCA_943734735.2) with minimap2 v2.17 [29]. Decontaminated reads were initially assembled with Hifiasm v0.19.8, following default parameters (S1 Fig) [30]. No duplication purging or polishing steps were performed on the resulting assembly, based on the recommendations by Hifiasm, the low error rate of PacBio sequencing and the high read coverage. The taxonomy profiles and GC content of the assembled contigs were analysed with SprayNPray v 1.0 [31] and an in-house database of microsporidian, bacterial and non-microsporidian eukaryotic genomes (*Anopheles gambiae* [GenBank: GCA_943734735.2], *Arabidopsis thaliana* [GenBank: GCA_000001735.2], *Brugia malayi*.[GenBank: GCA_000002995.5], *Danio rerio* [GenBank: GCA_049306965.1], *Drosophila melanogaster* [GenBank: GCA_000001215.4], *Enterocytozoon bieneusi* [GenBank: GCA_000209485.1], *Encephalitozoon cuniculi* [GenBank: GCA_000091225.2], *Escherichia coli* [GenBank: GCA_000005845.2], *Gallus gallus* [GenBank: GCA_016699485.1], *Hamiltosporidium tvaerminnensis* [GenBank: GCA_022605425.2], *Homo sapiens* [GenBank: GCA_000001405.29], *Mus musculus* [GenBank: GCA_000001635.9], *Saccharomyces cerevisiae* [GenBank: GCA_000146045.2], *Staphylococcus aureus* [GenBank: GCA_000013425.1], *Streptococcus pneumoniae* [GenBank: GCA_000019265.1], *Tribolium castaneum* [GenBank: GCA_031307605.1], *Vittaforma corneae* [GenBank: GCA_000231115.1], *Wolbachia pipientis* [GenBank: GCA_021496215.1], *Xenopus laevis* [GenBank: GCA_017654675.1]). Where further confirmation of ORF homology within outlier contigs was required, ORFs were predicted with NCBI ORFfinder [32] and ORFs exceeding 1 kb in size were examined with BLASTp. Sequencing depth of assembled contigs was calculated with samtools v1.16.1 ‘depth’ command [33]. After the prediction of potential tetraploidy of MB, Hifiasm assembly was repeated for the final genomewith ploidy set to 4 (--nhap option; S1 Fig). Again, no subsequent duplicate purging or polishing was performed after assembly.

A single Illumina sample was submitted, NGS library prepared with 9.1 µg of gDNA using the NEBNext Ultra II FS DNA Library Prep Kit for Illumina (New England Biolabs, Ipswitch MA, USA) and sequenced on a NextSeq2000 with 2 × 100 bp paired-end reads (Illumina Inc, San Diego CA, USA). The provided adapter sequences were trimmed with Trimmomatic v0.39 [34] and host reads were removed using the *An. gambiae* genome with Bowtie2 v2.4.2 [35].

Nanopore sequencing was prepared with 1.5 µg of HMW gDNA using a Ligation Sequencing Kit V14 and sequenced with a PromethION system (Oxford Nanopore Technologies plc, Oxford, UK). Data was provided by Edinburgh Genomics as FASTQ and BAM files for the raw reads and methylation base calls, respectively. The methylation BAM file was aligned to the final MB SOUVK7 assembly with Dorado v0.5.2 aligner [36], reviewed with Modkit v0.4.4 [37] summary function with ‘no filtering’ and the final bedMethyl file of base modification calls was generated with Modkit pileup using the “traditional” preset argument, which evaluates both 5mC and 5hmC instances in a single dataset. Methylation density was calculated across all chromosomes as the frequency of CpG methylation over total bases within 3,000 bp windows (S1 Fig).

### Gene prediction and genome annotation

Genome masking was performed with RepeatModeler v2.0.6 [38]and RepeatMasker v4.1.7 [39]) for repeat family identification and genome masking, respectively [40]. To ensure prediction of broader microsporidian repeat families, modelling was performed on all available chromosome-level and complete Microsporidia genomes of *Antonospora locustae* CLX, *Encephalitozoon cuniculi* GB-M1, *Hamiltosporidium tvaerminnensis* FI-OER-3-3, *Vairimorpha necatrix* (NCBI accession: GCA_007674295.1, GCF_000091225.2, GCA_022605425.2 and GCF_036630325.1, respectively) and the final assembly of Microsporidia sp. MB SOUVK7. Gene prediction and genome annotation was performed with Funannotate v1.8.17 [41] supplementing prediction with independently run Augustus v3.5.0 [42] and GeneMark-ES v2.5 [43] files. Augustus gene prediction was run specifying species as ‘encephalitozoon_cuniculi_GB’ and ‘intronless’ for gene model parameters due to the reported lack of introns in several Microsporidia genomes [44, 45], whilst GeneMark-ES was run following eukaryotic prediction (‘-euk’). Funannotate ‘predict’ function was run using the ‘microsporidia_odb10’ BUSCO database, whilst supplementing the previous prediction files and removing GlimmerHMM functionality from the pipeline, due to excessive gene fragmentation. Use of ‘microsporidia_odb10’ datasets were required necessary in all Funannotate pipelines as the most recent BUSCO ‘microsporidia_odb12’ had yet to be implemented into the Funannotate package at the time of analysis. InterProScan5 analysis [46] was run locally as part of the Funannotate ‘iprscan’ command, with resulting files being provided as part of ‘annotate’ command along with the ‘microsporidia_odb10’ BUSCO database.

Telomeric regions were identified from the results of RepeatMasker, verifying the presence of (TTAG)_n_ repeat regions at the ends of each chromosome. Repeat content along MB chromosomes were incorporated and visualized in Circos as custom BED files, using the shinyCircos-V2 R package [47]. Final genome integrity was assessed with BUSCO v5.4.5 against the latest ‘microsporidia_odb12’ dataset [48], as the current database was accessible through stand-alone BUSCO tools.

As ribosomal RNA (rRNA) prediction is currently not implemented into the Funannotate pipeline, rRNA prediction was performed with Barrnap v0.9 [49], specifying ‘bacteria’ for the kingdom parameter to account for the reduced size of microsporidian rRNAs. The resulting GFF file was integrated into the primary Funannotate output GFF with AGAT v1.4.1 ‘manage_IDs’ tool [50].

### Genome ploidy, gene orthology, and GO analysis

For comparison of MB SOUVK7 assembly quality to other published genomes, statistics of NCBI reference genomes were collected with a focus on assemblies of higher levels (“Complete”/“Chromosome”) and closely related species (S4 Table).

To initially assess the haploid assembly for potential polyploidy, chromosomal synteny was examined with MCScanX v1.0.0 [51]. Host-decontaminated reads of both Illumina and PacBio samples that mapped to the final MB assembly were also used to analyse ploidy (S1 Fig). For reference-free kmer analysis, GenomeScope v2.0.1 was run using 21 bp kmer length, and visualised with Smudgeplot v0.40, following default parameters [52]. Additional validation of predicted ploidy was provided with ploidyNGS v3.1.3 [53].

Predicted genes of MB SOUVK7 were further investigated to assess gene orthology, encoded-protein function and localisation, and genetic domains. Comparative orthology between MB SOUVK7 and all other NCBI available proteomes of Microsporidia (46 proteomes, S4 table) was assessed using OrthoFinder v2.5.5 [54], including *Mitosporidium daphniae* and *Amphiamblys sp.* acting as an outgroup as species of basal Microsporidia or ‘Microsporidia-like’ organisms [14, 45, 55, 56]. The resulting multi-gene phylogeny provided by OrthoFinder was visualised with TreeViewer v2.2.0 [57]. Examination of all to all BLASTp and tBLASTn search results confirmed the lack of orthologs in any species. BLAST predictions were only included into results if all BLAST-predicted orthologs were identified with an e-value > 1e^− 5^ and existed in an orthogroup containing all undetected orthologs. Protein function was predicted with stand-alone DeepLoc v2.0 [58] using the ‘accurate’ methodology. Additional genetic domains were predicted with standalone versions of TargetP v2.0 [59] and DeepTMHMM v1.0 [60], to predict signal peptides and transmembrane domains, respectively.

The spore wall and polar tube organelles of Microsporidia are compromised of several proteins unique to the phylum and so, can act as good bioinformatic targets to examine species taxonomy and infectious pathways. To examine the presence or absence of polar tube and spore wall proteins in MB SOUVK7 and broader Microsporidia, the orthogroup results of the above OrthoFinder analysis were searched against a pre-determined list of confirmed polar tube proteins (PTPs) and spore wall proteins (SWPs; S6 Table). Orthogroups with corresponding orthologs were compiled, with entries being concatenated when multiple search orthologs for the same protein family was provided and multiple orthogroups were found. Any consistent lack of orthologs across both MB genomes or Enterocytozoonida clade was verified through tBLASTn searches of all identified orthologs to the relative genomes.

Genes associated with methylation, telomeric and centromeric maintenance were previously reported in other Microsporidia families including the Unikaryonidae [21] and so potential orthologs in MB were identified to hits in *E. cuniculi* using BLASTp with an e-value threshold of 1e^− 5^. Given the reduced nature of the Encephalitozoonidae genomes, genes associated to gene ontology (GO) terms for telomere maintenance (GO:0000723), heterochromatin formation (GO:0031507), centromere complex assembly (GO:0034508) and methylation (GO:0032259) for non-Microsporidia species across the fungal kingdom (*Allomyces macrogynus*,* Mucor lusitanicus*,* Rozella allomycis*,* Saccharomyces cerevisiae* and *Schizosaccharomyces pombe*) were supplemented into the existing OrthoFinder dataset (NCBI accession: : GCA_000151295.1, GCA_001638945.1, GCA_000442015.1, GCA_000146045.2 and GCA_000002945.3, respectively). Orthogroups including any of the supplemented GO genes were extracted from the total orthogroup data frame, and potential microsporidian orthologs were examined.

## Results

PacBio sequencing of *An. coluzzii* ovaries infected with MB generated 766,153 reads with an average read length of 15.5 kb (max length: 44.5 kb). Total GC content showed two distinct read populations of approximately 31% and 45%, hypothesised as MB and host, respectively. Upon removal of host reads, 125,854 reads remained (16.4% of total reads) with a GC content of only ~ 31%, suggesting the 45% GC reads belonged to the host (S2 Fig. A & B). This reduction in reads had little effect on mean read length but led to a notable drop in maximum read length (15.3 kb mean length; 29.8 kb max length). Illumina sequencing provided a dataset of 105,381,324 paired reads with an average sequence length of 93.8 bases. After removal of host and contaminant reads, 15,795,162 reads remained and accounted for 15.0% of total reads.

**Fig. 1:**
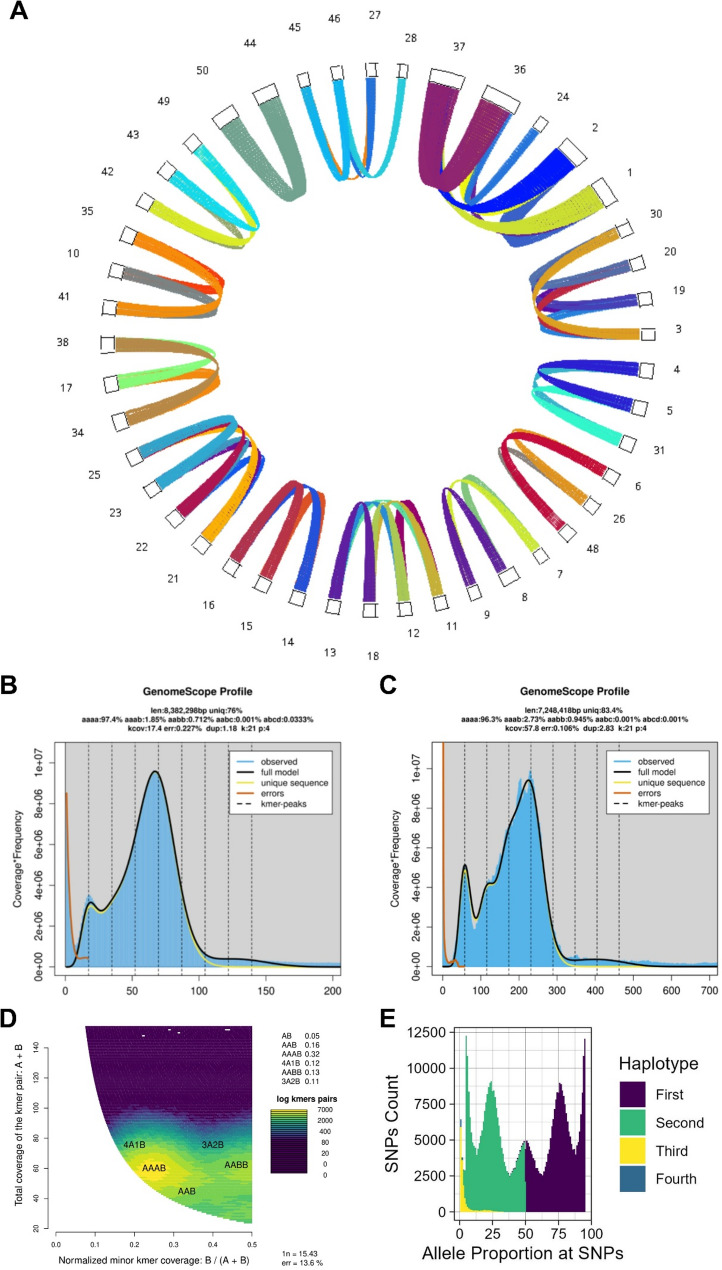
Predicting tetraploidy in Microsporidia sp. MB SOUVK7 through multiple analysis tools. **A** Chromosomal synteny of initial haploid assembly of MB SOUVK7, re-organised to highlight duplicate syntenic contigs. 13 distinct groups of syntenic contigs are identified with 2 to 5 contigs in all groups. The most abundant counts are 3 and 4 contigs encompassing 7 and 4 contig groups, respectively. Synteny analysis and visualisation was generated with MCScanX. **B-C **GenomeScope plots of Illumina and PacBio reads, respectively. Although both plots only exhibit 2 clear peaks, additional 2 kmer peaks are predicted between the observed peaks. Both profile statistics also confirm tetraploidy with 4-mer haplotype predictions, additionally suggesting autotetraploidy with both profiles having higher AAAB heterozygous abundance. **D** Smudgeplot heatmap visualisation of PacBio read data highlighting the significantly elevated abundance of 4-mer pairs (AAAB & AABB) compared to other kmer values within the data. **E** Histogram of ploidyNGS results from PacBio read data, plotting distribution of reads associated to SNP haplotypes across the genome. The frequency of various haplotypes observed across all aligned reads at each SNP position are calculated as a percentage of total haplotypes (X-axis). Where multiple haplotypes are observed at the same SNP, haplotypes are ordered from ‘First’ to ‘Fourth’ in order of descending proportion. All SNPs exhibiting the same haplotype percentages are counted and plotted as abundance (Y-axis), with colour showing representing haplotypes. The five peak distribution of the plot at 5%, 25%, 50%, 75% & 95% clearly correlate to exemplary tetraploid results provided with the tool [53]

A preliminary assembly of non-host PacBio reads using Hifiasm in default diploid mode yielded 77 contigs, 56 of which were assigned to Microsporidia by taxonomic profiling (S2 Fig). Plotting of this assembly showed 13 clusters of duplicated contigs, strongly suggesting that the contigs were incorrectly phased and likely due to genome polyploidy (Fig. 1.A). GenomeScope analysis of both Illumina and PacBio reads predicted a tetraploid kmer distribution with significant bias towards AAAB kmers over AABB kmers, and predicted haploid genome sizes of 8.3 and 7.2 Mb, respectively. Smudgeplot outputs also supported tetraploidy with a majority prediction of AAAB kmers in Illumina reads (Fig. 1.D).

Reassembly of non-host reads assuming tetraploidy generated 20 contigs, with SprayNPray separating them into two distinct populations; a dense clustering of 14 contigs (GC content: ~31%, read depth: ~175-225x) and a sparser cluster of 6 contigs (GC content: 39-56.5%, read depth: ~3-13x) of microsporidian and bacterial origins, respectively (S2 Fig. D). BLAST searches of the bacterial contigs again found strong homology to sequences of C*edecea neteri* and Circoviridae-like viruses. It should be noted that these bacterial contigs were similarly identified in the initial assembly. Of the 14 remaining microsporidian contigs, one contig was identified as an outlier due to its lack of telomeres, significantly reduced size and elevated read depth (9.4 kb and ~ 245x, respectively). The GC content of this outlier was also found to be higher than all other microsporidian contigs at 35.6% GC compared to a mean of 31.2% in all other contigs. Visual inspection of the contig confirmed a lack of telomeric repeats, whilst BLASTp searches of predicted ORFs within the contig identified 6 sequences associated with transposable elements in Microsporidia and broader fungal species, including LINE1 transposable elements, RNA-directed polymerase and reverse transcriptase. Additionally, no homologs to mitochondrial genes were identified in this contig. Based on the inconsistent characteristics of this contig to all other microsporidian contigs and the highly repetitive nature of predicted ORFs, this contig was excluded from the final genome as an artifact of genome assembly.

**Fig. 2:**
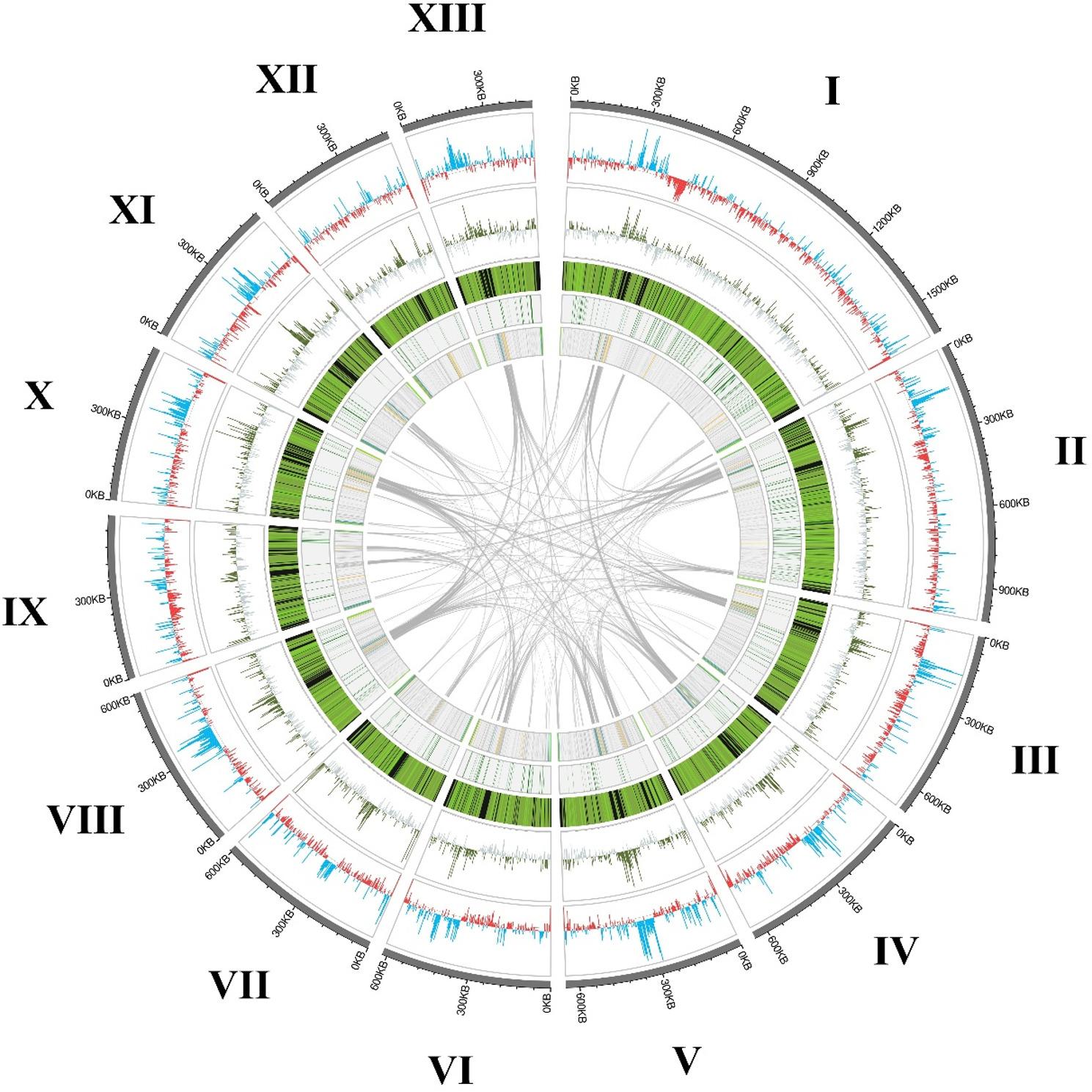
Circos plot of all chromosomes of Microsporidia sp. MB SOUVK7, highlighting genetic characteristics. The lengths of the 13 chromosomes are represented by their respective segment, with circles representing different analytical datasets. Outer circle shows local GC content across chromosomes as the mean across a 3,000 bp window. Cyan and red peak colours illustrate the windows GC content being above or below the mean GC content of the assembly (31.13% GC), respectively. The second circle plots peaks of methylation density as total CpG bases per 3,000 bp window. Green peaks represent windows of CpG density above the assembly mean (0.01306 CpG/base), whilst grey represents windows below mean. The third circle demonstrates the local gene density within the chromosome, calculated as the number of gene annotations per 3,000 bp window. Density is shown as a heatmap with black denoting zero genes per window, with continuous green gradient for windows with greater than one gene per window. The fourth circle represents the location of BUSCO genes across the genome with green, blue and red points representing complete, duplicated and fragmented genes respectively. The inner circle highlights the location of various retroelements, coloured by retroelement groups determined by RepeatModeler (cyan: long interspersed nuclear elements [L1, RTE-X, RTE-BovB], orange: long terminal repeats [Ty3/Gypsy], dark grey: long interspersed nuclear elements [Dong-R4]; on a light grey background)

**Fig. 3:**
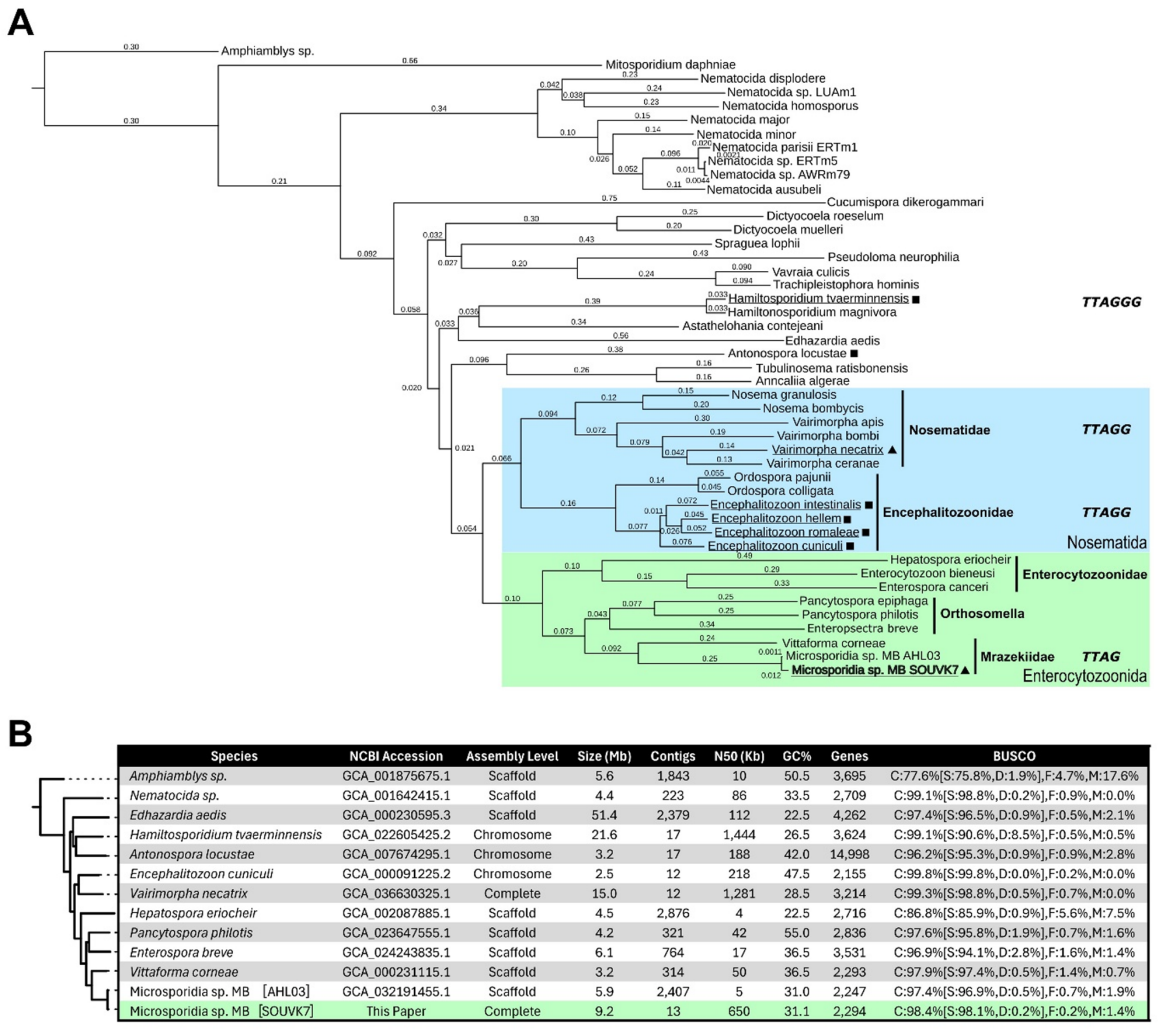
Comparative phylogeny and assembly statistics of Microsporidia sp. MB SOUVK7 to reference genomes of the Microsporidia phylum. **A** Multi-gene phylogeny of the Microsporidia sp. MB SOUVK7 in the context of the Microsporidia phylum. Microsporidia sp. MB SOUVK7 is indicated in bold. Coloured boxes and vertical lines highlight clades and families of related species, respectively, as per [14]. Solid square and square symbols indicate species possessing chromosome and complete level assemblies, respectively. Underlined species assemblies include telomeric kmer sequences, with encoded kmers in bold italics to the right of the respective species. Phylogeny was generated as part of the OrthoFinder pipeline and the tree was visualised with TreeViewer v2.2.0 [57]. Microsporidia sp. MB SOUVK7 localises closely the MB AHL03 within the Mrazekiidae, a family within the Enterocytozoonida clade. **B** Comparison of the assembly characteristics for Microsporidia sp. MB SOUVK7 against other pertinent microsporidia species. Tree was generated from a multi-gene phylogeny generated by OrthoFinder, with *Amphiamblys* sp. and *M. daphniae* as the outgroup and branch numbers representing branch length (substitutions per site). Table values are collected from NCBI with BUSCO scores being calculated for total nucleotide sequence with the ‘microsporidia_odb12’ dataset

The finalised genome had a total size of 9,162,986 bp (9.16 Mb) across 13 complete chromosomes with an N50 of 650,044 bp (Fig. 2). This was broadly consistent with the genome sizes predicted by GenomeScope of 8.4 Mb and 7.2 Mb during the ploidy analysis of PacBio and Illumina reads, respectively. Based on this final assembly, PloidyNGS plots also supported a tetraploid genome, with five clear peaks across individual and consensus plots of all chromosomes, consistent with example tetraploid data (https://github.com/diriano/ploidyNGS/blob/master/test_data/ploidyNGS_results/DataTestPloidy4.tab.ExplorePloidy.png). Chromosome length ranged from 477 kb to 1.6 Mb with a mean coverage of 208 × (184–225x) and mean GC content of 31.13% (30.79–31.74%). Illumina reads mapped against the genome with a mean depth of 172.7 × (170.1–177.4x). Funannotate and Barrnap predicted a total of 2,435 genes distributed across all chromosomes (0.78 genes per 3 kb, 0.69–0.91), comprising 2,294 protein-encoding genes, 87 tRNAs and 54 rRNAs (Fig. 2). Repeat sequences were predicted across 40.66% of the genome. BUSCO analysis reported similar assembly completeness to other Enterocytozoonidae species with 98.4% of complete genes and 1.4% missing genes (Fig. 3). The multi-gene phylogeny provided by OrthoFinder also placed Microsporidia sp. MB SOUVK7 within the Enterocytozoonida clade and Mrazekiidae family, in immediate relationship to Microsporidia sp. MB AHL03 and *Vittaforma corneae* (Fig. 3).

During the masking step of assembly, RepeatMasker identified 40.66% of the genome as repeat sequences, predominantly as retroelements and unclassified repeats accounting for 26.17% and 6.73% of the total genome, respectively. The most abundant of these retroelements were Dong-R4 type long interspersed nuclear elements (LINEs) which evenly distributed within and between chromosomes (Fig. 2). The remaining LINEs and long terminal repeats (LTRs) largely localised at the termini or discrete regions in the centre of chromosomes. Terminal regions of all MB chromosomes were found to possess simple 4-mer telomeric motifs of TTAG. Telomere repeat regions varied in size between 1,312 bp and 7,145 bp, with a mean length of 3,900 bp and were often flanked by further LINE repeat groups. Telomeric regions exhibited significantly lower frequencies of methylated cysteines when compared to mean CpG methylation of the whole assembly (0.00326 vs. 0.01306 CpG/base, respectively). Repeat-rich areas within the chromosomes, comprised mostly of L1 type LINEs and Ty3 type LTRs, spanned large central sections  (30-82 kb) of most chromosomes. Synteny and BLAST analyses of these repeat-rich regions showed low synteny conservation between chromosomes, however all regions consisted of conserved repeat motifs uniquely organised into each region. These regions were consistently the areas of highest local GC content within chromosomes (47.6% Mean local GC, 2.2% StDev). The high density of repeats was also reflected by decreases in local gene density. Although methylation was observed across the entire genome, these regions correlated with high CpG methylation (0.02399 vs. 0.01306 mean CpG/base for high methylation regions and whole assembly, respectively). Most chromosomes contained one example of these high GC, high methylation, and low gene density regions, however chromosomes II and VI potentially displayed pairs or splits in these regions. Gene annotation of SOUVK7 predicted 2,294 protein-encoding genes, and was comparable to other members of the Enterocytozoonida clade, but remained low for the broader phylum (Fig. 3).

Different signal prediction tools identified between 133 and 462 genes with signal peptides and a total consensus of 12 genes, with DeepLoc and TargetP providing largest consensus of 160 genes (S5 Table). Similarly, comparison of transmembrane region (TMR) prediction tools showed a closer consensus to one another of 424 genes (18.7% genes; 521 & 430 predictions for DeepLoc and DeepTMHMM, respectively). Over 80% of gene products were predicted by DeepLoc as intracellular, whilst only 151 and 43 gene products are predicted to be extracellular or transmembrane (6.6% and 1.9%, respectively). Of the 2,294 protein-encoding genes of MB SOUVK7, 2144 (93.5%) are assigned to microsporidian orthologies with MB SOUVK7 appearing in 16.7% of all orthogroups and containing only 2 species-specific orthogroups. Furthermore, 1998 MB SOUVK7 genes were found to have at least one ortholog in MB AHL03, accounting for 95.6% of orthogroup-allocated genes. Based on these parameters and total gene counts, gene predictions of MB SOUVK7 and MB AHL03 were broadly considered homologous, as reflected in the resulting multi-gene phylogeny (Fig. 3).

Homology searches for telomeric and heterochromatin maintenance components (GO:0000723 & GO:0034508, respectively) identified 24 MB orthologs of the 38 reported genes from *E. cuniculi*. Within the telomere maintenance machinery, only telomerase reverse transcriptase and the Rad32-Rad50 DNA-repair protein complex was found in MB, accounting for only 25% of *E. cuniculi* components. Notably, MB lacked components of both the Cdc13-Stn1-Ten1 and shelterin complexes, associated with protecting against telomeric degradation despite well-conserved motifs still being seen in all chromosomes. OrthoFinder searches for broader fungal GO components identified two additional orthologs of telomeric maintenance proteins in MB (Rad51 & Rad52) [61, 62]. Although no components of ‘centromere complex assembly’ GO term (GO:0034508) were previously reported in Microsporidia [21], OrthoFinder found four associated orthogroups including hits for all species, including MB. Orthogroups were found to contain complex ATPase subunit (*INO80*) and chromatin segregase (*YTA7*), histone chaperone (*MIS16*) and monopolin (*MAM1)* genes associated with centromere remodelling, histone chaperoning, and meiotic regulation, respectively.

MB orthologs for the ‘heterochromatin formation’ GO term (GO:0031507) demonstrated the largest divergence from previous *E. cuniculi* predictions. BLAST searches only found 21 MB ortholog genes to the 30 *E.* cuniculi genes assessed. Most notably, MB only possessed three of five components of the Clr4 methyltransferase complex (CLRC) required for heterochromatin formation (Swi6, Cul4 and Rik1), of which all five components were reported to be essential for CLRC function [63]. MB encoded orthologs for the four core histone proteins (H2A, H2B, H3 & H4) and, mostly interestingly for centromere protein (CENP-A), a highly divergent variant of H3 histones most commonly associated with epigenetic mechanisms in the centromere [64]. Finally, all methylation components in the Unikaryonidae were found in MB including all 3 paralogs of rRNA m5c methyltransferase, with the exception of nucleolar essential protein 1 (NEP1) associated with rRNA SSU methyltransferase.

**Fig. 4:**
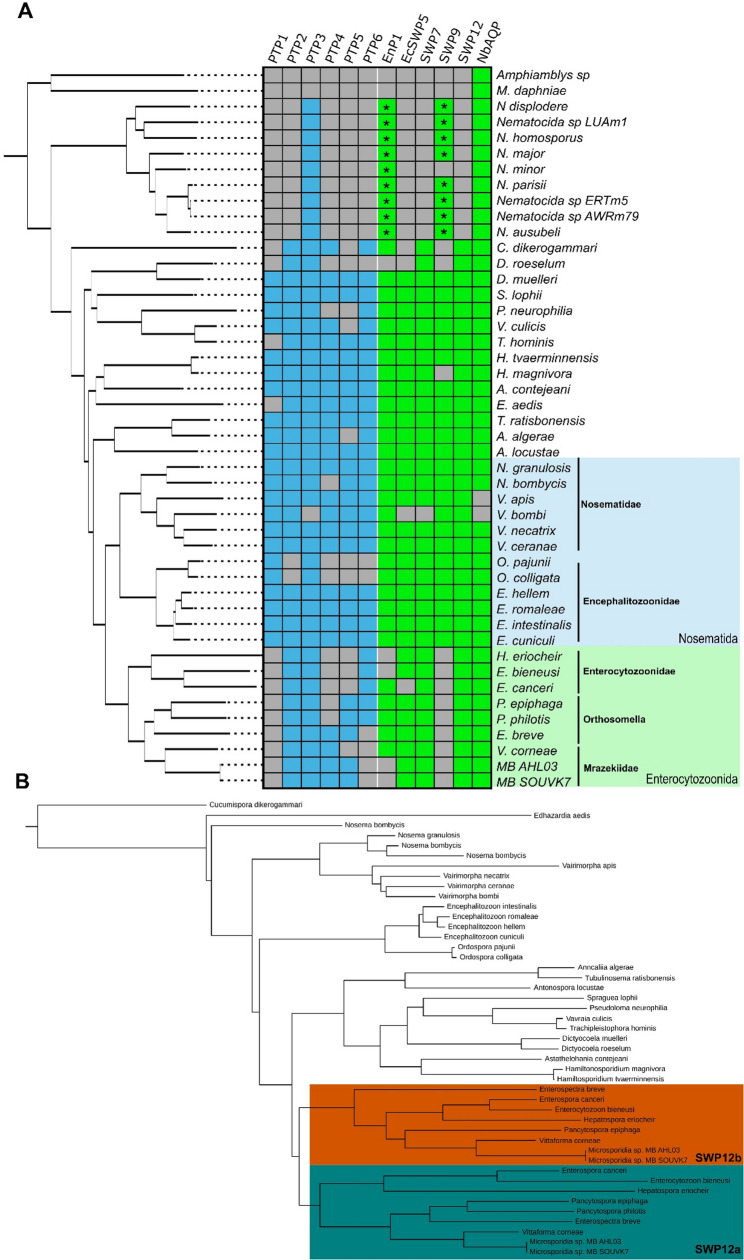
**A** Orthology of principal polar tube and spore wall proteins across the Microsporidia phylum. Phylogeny of all analysed species are illustrated to the left of the table and is based on the total species phylogeny generated by OrthoFinder. Species in possession of at least one ortholog of the associated polar tube and spore wall proteins are represented by blue and green colours, respectively. Species lacking any orthologs are illustrated by grey. Orthologs denoted with a star represent *Nematocida* genus orthologs first identified through BLASTp searches and verified to exist in a single orthogroup. **B **Phylogeny of orthogroup associated with spore wall protein 12. General phylogeny of the group follows the multi-gene phylogeny of the phylum (Fig. 3), with a branch duplication at the Enterocytozoonida node. The two distinct isoforms are highlighted in green and gold for SWP12a and SWP12b, respectively

Spore wall proteins (SWPs) and polar tube proteins (PTPs) play essential roles in interaction and infection of host tissues. MB SOUVK7 possessed several proteins associated with the spore wall and polar tube infectious machinery. OrthoFinder results provided at least one Microsporidia orthogroup for 19 of the 24 SWPs and PTPs examined (S5 Table), with the remaining 5 search proteins being unassigned or only existing in single species orthogroups. An additional 7 protein groups (PTP7, SWP1, NbSWP5, SWP8, SWP25, SWP26, and SWP30) lacked species diversity outside of the Nosematida clade so were removed from the final results. The remaining protein families included 6 of each of the PTPs and SWPs with extensive coverage across the phylum (Fig. 4.A), including endospore protein (EnP1) [65]. No orthologs were identified in the outgroup species of *M. daphniae* and *Amphiamblys sp.*, with the exception of aquaporin (NbAQP) which was found in all species except *Vairimorpha apis* and *Vairimorpha bombi*. The extreme fragmentation of these two genomes may have contributed to this lack particularly when compared to other genomes within the genus. Furthermore, *Nematocida* species possessed orthologs for only PTP3, EnP1 and SWP9, with further tBLASTn searches verifying the lack of all other gene families. Due to the basal nature of the *Nematocida*, this lack of additional SWP and PTP orthologs may have highlighted the limitations of OrthoFinder to identify orthologs within highly divergent species. This was shown as additional BLASTp searches were required to initially identify orthologs for EnP1 and SWP9, however the lack of detection of other gene orthologs may have been due to a lack of resolution in the phylogeny at the base of the phylum.

Within the Enterocytozoonida clade, total loss of PTP1, PTP4 and SWP9 orthologs was observed across all 9 species including MB SOUVK7, despite near complete representation within all other species of the phylum. PTP4 and PTP5 families were both allocated to the same orthogroup, with one MB ortholog (ACK3DK_000951) allocated to the PTP5 family, due to its taxonomic position. As no orthogroups associated with PTP4 and PTP5 included any predictions for *V. corneae*, extra orthogroups for previously determined orthologs (VICG_01195 and VICG_01807, respectively) were further investigated [66]. The two orthogroups of PTP4 and PTP5 only included *V. corneae* orthologs and complimentary MB orthologs, suggesting a potential misidentification of the PTP orthologs in prior literature or prediction of an alternative PTP family uniquely associated to the Mrazekiidae family.

OrthoFinder also identified orthologs of 4 SWP families (EcSWP5, SWP7, SWP12 and NbAQP) in both MB genomes, whilst reporting loss of SWP9 and EnP1. All SWP9 orthologs correlated to a single orthogroup and included all remaining species except all Enterocytozoonida species. Further tBLASTn searches confirmed these losses with no significant hits, supporting the family wide loss of this gene. No EnP1 orthologs were identified in either MB genome despite the detection of orthologs for all other Mrazekiidae species and this was also confirmed with no significant tBLASTn hits to either genome. The lack of EnP1 was not unique to MB however as orthologs were also missing in *Enterocytozoon bieneusi*, *Hepatospora eriocheir*, *Dictyocoela roeselum* and the entirety of the *Nematocida* genus.

Searches for SWP12 presented two MB distinct paralogs within the same orthogroup whilst only single orthologs were identified across the phylum. This orthogroup also included both SWP12 paralogs for all species of the Enterocytozoonida clade, but tBLASTn searches still failed to identify secondary paralogs across the rest of the phylum (Fig. 4.B). The resulting orthogroup tree followed the standard Microsporidia phylogeny and showed a clear duplication event, resulting in the two Enterocytozoonida paralogs, occurring at the basal node of the family [19]. Both branches mimicked that of the expected family phylogeny except for a single SWP12a ortholog in *Pancytospora. philotis*. The comparison of taxonomic lengths between both branches suggested a closer conservation of the complete branch to the wider protein family, leading to the tentative naming of SWP12a and SWP12b to the complete and partial branches, respectively. Alignment of the paralogs showed strong conservation along the lengths of both genes, however SWP12a appeared to possess an additional 8–15 amino acids at the N-terminus, likely contributing to much of the phylogenetic separation of the paralogs.

## Discussion

Despite the growing number of Microsporidia reference genomes on NCBI, very few are resolved to a chromosome or complete level (53 reference genomes with 6 at chromosome-level and 1 at complete level, as of Feb 2026). Furthermore, the taxonomic variety of these genomes is very limited with five of the seven belonging to the Nosematida clade. Through multi-platform sequencing, we have effectively expanded upon existing genomes of Microsporidia sp. MB and the broader Enterocytozoonida clade with one of the first telomere-to-telomere, complete genome assembly in the clade. This novel complete assembly of MB SOUVK7 expands and diversifies current genomic datasets into the Mrazekiidae family, whilst providing further insight into unique genetic characteristics of MB. Although the Microsporidia phylum exhibits a wide range of genome sizes (1.52–51.35 Mb, *Alternosema astaquatica* and *Edhazardia aedis* respectively), the 9.16 Mb genome of MB SOUVK7 represents a notable increase in genome size within the Enterocytozoonida clade, which have previously reported sizes of 6 Mb or lower (Fig. 3). This includes a near 2-fold size increase compared to the 5.9 Mb assembly of Microsporidia sp. MB AHL03 whilst reducing contig number from 2335 to 13 [24, 26]. Despite this increase in size, the predicted number of open reading frames (ORFs) was similar to that of closely related genomes, which suggests that much of the size increase may be attributed to the correct assembly of intergenic repeat regions associated with longer reads. This is supported by a prediction of 28.2% interspersed repeats in MB SOUVK7, compared to previous mean percentage repeats of 0.57% and 7.32% reported in the Enterocytozoonida clade [24, 26, 67]. The MB SOUVK7 genome also contains a shift in proportion of transposable elements away from DNA transposons (2.03% and 5.83%) and towards LINEs (24.40% and 0.34%, % assembly for MB SOUVK7 and mean Enterocytozoonidae). Although longer and more abundant LINE TEs are revealed by complete assembly, it is also possible that the symbiotic nature and principally vertical transmission of MB may have some influence on the evolution and size of the MB genome [10, 25, 68]. Vertical transmission and asexual reproduction often lead to strong genetic bottlenecks, resulting in expansions of LTR and LINE transposable elements within a population. This could explain genome size expansion in several vertically-transmitted Microsporidia [67, 69]. Alternatively, the expansion in transposable elements could be the result of relaxed selection and the inability to eliminate mildly-deleterious transposable element insertions.

Flanking sequences of MB chromosomes provide further evidence of telomeric evolution across the Microsporidia with the TTAG motif observed in MB SOUVK7 representing the most reduced repeat in the phylum. Two guanine deletion events within the telomeric regions are evident across the phylum, firstly at the node shared with *Hamiltosporidium* species (TTAGGG to TTAGG) and secondly at the node shared with the Nosematida clade (TTAGG to TTAG). The recently released genome of *Glugoides intestinalis* (in Glugeidae family, Enterocytozoonida clade) also shows a 4-mer TTAG repeat motif, further supporting telomeric motif reduction within the Enterocytozoonida clade [18, 70]. No telomeric motifs are reported for *A. locustae*, despite its chromosome level assembly, which could otherwise better localise the taxonomic position for the first reduction event [22]. Telomeric simple repeats in MB SOUVK7 are also flanked by short stretches of LINE repeats of up to 10 kb in the majority of chromosomes. In *Encephalitozoon* assemblies, telomeric CpG methylation is more frequent than in the rest of the genome, but the reverse is seen in MB SOUVK7 [21]. Synteny analysis shows no evidence of subtelomeric regions, which are characterised by mosaic collections of interchangeable segmental duplications (EXT repeats), despite their detection in all *Encephalitozoon* species [71]. Although subtelomeres appear to be lacking in MB, the presence of highly repetitive LINEs adjacent to the telomeric regions may echo subtelomeric characteristics without the broader homology between chromosomes.

In addition to telomeres, all 13 chromosomes exhibit distinct regions of differential genomic architecture, with several characteristics correlating with centromeric regions in fungi and broader eukaryotes. These regions are at least 10 kb from chromosome ends and are gene sparse and GC rich whilst possessing dense clusters of CpG methylation and specific retroelements. These regions were identified by localisation of non-R4 LINE and LTR retroelements, which only represent approximately 3.7% and 3.4% of total retroelement predictions. Despite lacking variety, these elements still represent 18.7% of bases associated with repeats. These repeat clusters have knock-on effects on local GC content and gene density. Ty3 LTRs are found almost exclusively in these regions and have been identified within centromeres of plants and fungi [72, 73]. Microsporidian Ty3’s have, however, been found to have diversified dramatically within the phylum with the typical two ORFs of Pol reverse transcriptase and Gag structural protein being amalgamated into a single polyprotein [74]. The nearby presence of large rRNAs to these distinct regions in MB SOUVK7 may mimic the roles of long non-coding RNAs (lncRNAs), similar to mammalian and fungal centromeres [75]. Changes in local GC content can also define centromeres, however in fungal centromeres and similar regions in *A. locustae*, a local reduction in GC content is seen contrary to the increase observed in MB [75–77]. In MB SOUVK7, the GC content of these regions is actually higher than or equal to the GC content of other microsporidian genomes, with some MB regions having up to 49.5% GC. It must be noted however, that total mean GC content of both *A. locustae* and Dikarya fungi are considerably greater than that of MB (41.6% and 40–65%, compared to 31.13% respectively), which may limit the biological feasibility of further GC reduction in MB [22, 72].

Finally, Dorado analysis of these regions consistently demonstrate elevated CpG methylation compared to the rest of the chromosome. The prediction of many methylation-associated proteins in MB supports the functionality of the CpG methylation pathways. Highly localised methylation is observed in centromeric regions of fungi, plants and animals, supressing gene expression and assisting CENP-A and H3 nucleosomes with centromeric chromatin formation [77]. Methylation of microsporidian chromosomes has only been examined in the Encephalitozoon genus (Unikaryonidae family), showing elevation in the telomeric and sub-telomeric regions, however this may not be comparable due to their lack of regional centromeres [21]. Within the broader fungal kingdom, elevated CpG methylation is colocalised with the peripheries of sites of CenH3 (CENP-A ortholog) binding in *Neospora crassa* and *Vesticillium dahliae*, whilst also correlating with low local gene density and elevated repeat density [72, 78]. Although more data is required to confirm the final positions of centromeres in any microsporidian species, we note that the regions of interest highlighted by this study exhibit many of the characteristics of centromeres observed in broader eukaryotes, and hypothesize some microsporidia species may possess classical centromeres, instead of the point centromeres previously reported in the Unikaryonidae family [21].

Predictions of MB tetraploidy agrees with the prior association of high ploidy species with vertical transmission and insect infection [69, 79]. In a recent ploidy screen of 16 species, tetraploidy was identified in 6 species from a broad range of families (Neopereziidae, Glugeidae, Nosematidae and Enterocytozoonidae), whilst still possessing other diploid members in their respective families. Although not asserted in the ploidy screen, all 6 tetraploid species are known to infect a variety of arthropod hosts, whilst 8 of the remaining diploid species infect hosts of other phyla. This association is consolidated by the tetraploid characterisation of *V. necatrix*, and multiple novel uncharacterised assemblies from arthropod hosts exhibiting polyploidy (14 of 19 assemblies) [76, 80]. All ploidy analysis demonstrates a clear favoured kmer distribution of AAAB in MB, which also correlates with previously analysed Microsporidia species, supporting the broader trend of the phylum. Furthermore, predicted proportions of these kmer patterns in MB were found to be within the lower end of predicted values across the Microsporidia, suggesting relatively low levels of heterozygosity. This may stem from genetic bottlenecks associated with the primarily vertical transmission of MB, forcing populations within individual hosts to become relatively homogenous.

The proteins of microsporidian polar tube and spore wall have been shown to play important roles in both colonising and manipulating the host cells [1, 66, 81]. Although some protein families are well conserved throughout the phylum, other SWPs and PTPs have been found to be unique to specific genera and families. Given the loss and reduction of metabolic genes within the Enterocytozoonida clade, a potential minimalization of infection machinery was examined within the clade [19]. Our analysis found loss of PTP1, PTP4, PTP6, EnP1 and SWP9 in MB SOUVK7. The family-wide lack of PTP1 and PTP4 orthologs are surprising given their integral roles in polar tube adhesion to the host cell and previous electron micrographs demonstrating typical polar tubes within MB spores [25, 81, 82]. Notably, PTP1 and PTP4 both associate closely to the paralogous proteins of PTP2 and PTP5, respectively, and likely stem from recent gene duplication events [82–84]. As the presence of PTP2 proteins and polar tube functionality has been experimentally confirmed in the Enterocytozoonidae family, this may suggest reduction events of PTP1 and PTP4 within the wider Enterocytozoonida clade whilst the remaining paralogs have retained organelle functionality. Finally the loss of PTP6 in the Mrazekiidae family marks another reduction in polar tube components, however the primarily vertical lifecycle of MB may limit the necessity for a diverse variety of host invasion proteins [25, 68, 85]. SWP9 was also found to be lacking in all species of the Enterocytozoonida clade, mirroring the distribution of PTP1 and PTP4. SWP9 has been found to interact directly with the polar tube and PTP1 specifically, potentially as a polar tube anchoring component [86]. Given the proposed loss of PTP1 through gene reduction, the loss of SWP9 is feasible due to functional redundancy.

Whilst MB SOUVK7 exhibits many examples of gene redundancies, SWP12 was found to be a potential instance of gene diversification with two in-paralogs being observed across the Enterocytozoonida clade. Similar to SWP9, SWP12 contains a Bin/Amphiphysin/Rvs (BAR) domain, enabling host cell adhesion through GAG-binding [86, 87]. SWP12 may also assist in parasitism beyond adhesion, playing roles in lipoprotein binding, nutrient sequestering and vacuole formation during merogony and sporogony [88]. The functional overlap of SWP12 may have enabled SWP9 and EnP1 gene reduction without impacting parasitic pathways or MB retention in the host. As all experimental data for SWP12 is currently in *Nosema bombycis*, further characterisation of these predicted paralogs within the Enterocytozoonida clade may clarify their function further elucidate any deviations in SWP12 function.

With the recent description of MB and its potential as an agent for malaria control, the Enterocytozoonida clade are receiving renewed attention [10, 68]. As one of the first complete genomes within the Mrazekiidae family, the release of the MB SOUVK7 assembly expands our understanding of genomic architecture within the Enterocytozoonida clade and may act as a complete reference for population studies. MB has been identified in multiple African countries and several *Anopheles* species, with phenotypic differences already being noted between populations [9, 11, 12, 25, 68]. Further sequencing and assembly of complete MB genomes, whether *de novo* or assembled against MB SOUVK7, will better highlight genomic variance between populations and identify genes associated with host tropism, regional diversity and *Plasmodium* inhibition. Other mosquito-infecting Microsporidia species, including *Vavraia culicus*, have previously been considered as agents for malaria control, however high levels of host pathogenicity during horizontal transmission have limited their potential for environmental dispersion and retention [89]. As one of the only Enterocytozoonida species to exhibit a primarily vertical lifecycle, genetic comparisons of MB to species in the wider family may also highlight genes and pathways that enable this lifestyle [89]. The near-symbiotic life cycle of MB with anopheline mosquitoes is a crucial factor in its suitability as a control agent and so better understanding its mechanisms for limiting host parasitism may further help in developing MB dispersal protocols. 

## Supplementary Information

Supplementary Material 1. Table S3: Tabulated output of RepeatMasker run against Microsporidia sp. MB SOUVK7.

Supplementary Material 2. Table S4: Microsporidia assemblies included in the comparison on Microsporidia sp. MB SOUVK7 against the rest of the phylum. M. daphniae and Amphiamblys sp. was included as outgroups as a basal species of the Microsporidia and Cryptomycota, respectively.

Supplementary Material 3. Table S6: List of predicted orthologs to polar tube and spore wall proteins across species of the Microsporidia, as illustrated in Fig 4.

Supplementary Material 4. Fig S1: Table presenting the data from three sequencing platforms and their respective uses in the analysis pipelines presented in the methods . Crosses indicate the use of a platforms data in the analysis pipeline, whilst pipelines with multiple crosses were run independently for each dataset.

Supplementary Material 5. Table S5: List of signal peptide and transmembrane domain prediction of genes in Microsporidia sp. MB, including consensus gene counts between predictive tools.

Supplementary Material 6. Fig S2: Overview of reads and assembly of PacBio sequencing dataset before and after host read decontamination, and for haploid and tetraploid assembly conditions. A. FastQC overview of Raw PacBio read distributions of read count against read GC content. Two distinct peaks are visible of approximately 300,000 and 420,000 reads with GC content of 31% and 45%, respectively. B. FastQC overview of PacBio reads after removal of reads aligned to An. gambiae, exhibiting only a single peak of approximately 300,000 reads and 31% GC content. C. SprayNPray output figure of contigs assembled from host decontaminated reads following haploid assembly. The predicted phylogenetic origins of contigs are illustrated through point colour, with uncharacterised and bacterial contigs being clustered with low read depth and high GC content. Contigs for further syntenic alignment were isolated for the elongated cluster of 55 contigs with a GC content of ~31% and >40x sequencing depth. D. SprayNPray output figure of contigs assembled from host decontaminated reads following tetraploid assembly. The predicted phylogenetic origins of contigs are illustrated through point colour. The outlier contig that was excluded from the final genome is indicated with a black arrow.

## Data Availability

The datasets supporting the conclusions of this article are included within the article (and its additional files) and available in the NCBI BioProject repository, [PRJNA1200702]. The final assembly sequence of Microsporidia sp. MB SOUVK7 and associated feature files were submitted to NCBI WGS database under the accession . All raw Illumina, PacBio and Oxford Nanopore Technologies read data generated in this study were submitted to the NCBI Sequence Read Archive (SRA; https://www.ncbi.nlm.nih.gov/sra) under accession numbers: SRR32024117, SRR32024118 and SRR35190449, respectively.

## Abbreviations

GO: Gene orthology
HMW: High molecular weight
LINE: Long interspersed nuclear elements
lncRNA: Long non-coding RNA
LTR: Long terminal repeats
MB: Microsporidia sp. MB
ORF: Open reading frame
PTP: Polar tube protein
rRNA: Ribosomal RNA
SWP: Spore wall protein
TMR: Transmembrane region
