# Supplementary figures and images for: Telomere-to-telomere genome assembly of Microsporidia sp. MB, a microsporidian symbiont of Anopheles coluzzii isolated from Burkina Faso
Source: BMC Genomics. 2026 Apr 9;27:468. doi: 10.1186/s12864-026-12799-4 (PMC13174016; doi:10.1186/s12864-026-12799-4)

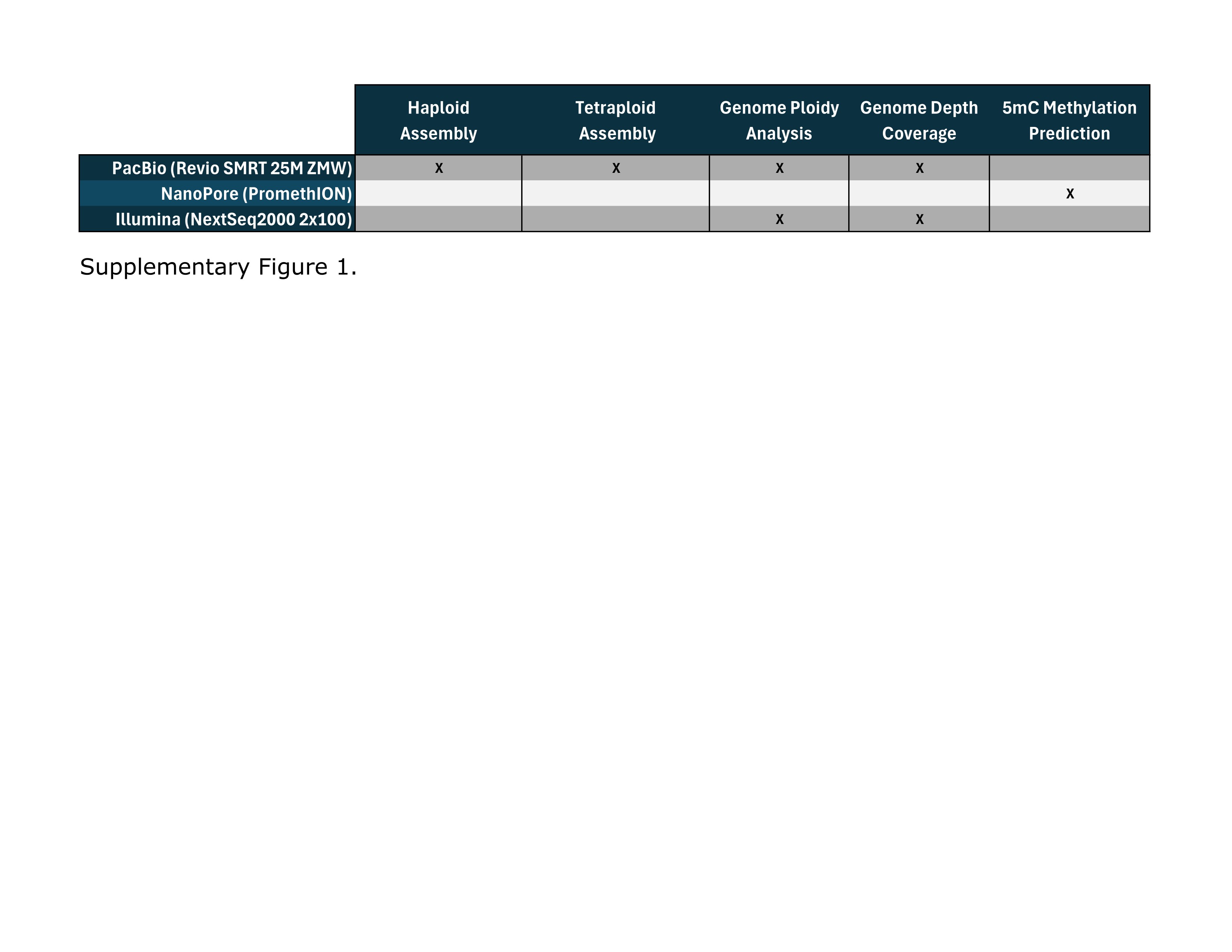

Supplement: Supplementary file 4 — Supplementary Material 4. Fig S1: Table presenting the data from three sequencing platforms and their respective uses in the analysis pipelines presented in the methods . Crosses indicate the use of a platforms data in the analysis pipeline, whilst pipelines with multiple crosses were run independently for each dataset. [file 12864_2026_12799_MOESM4_ESM.jpg]

## A. Per sequence GC content

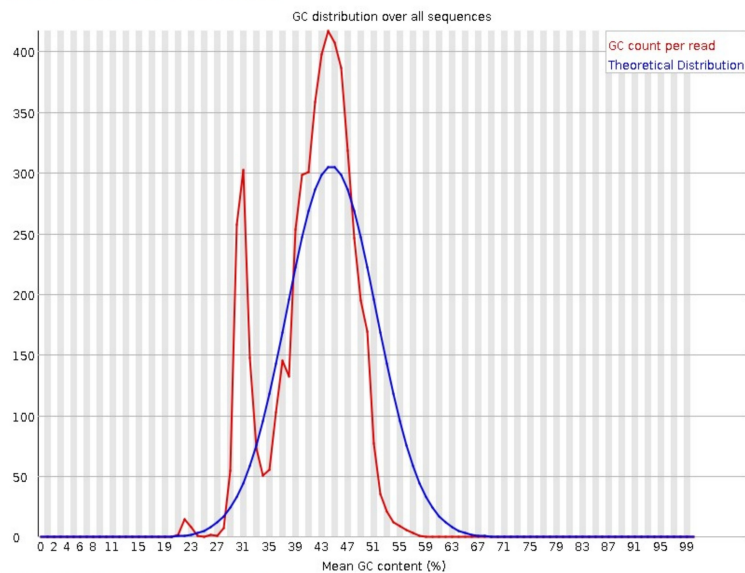

## B. Per sequence GC content

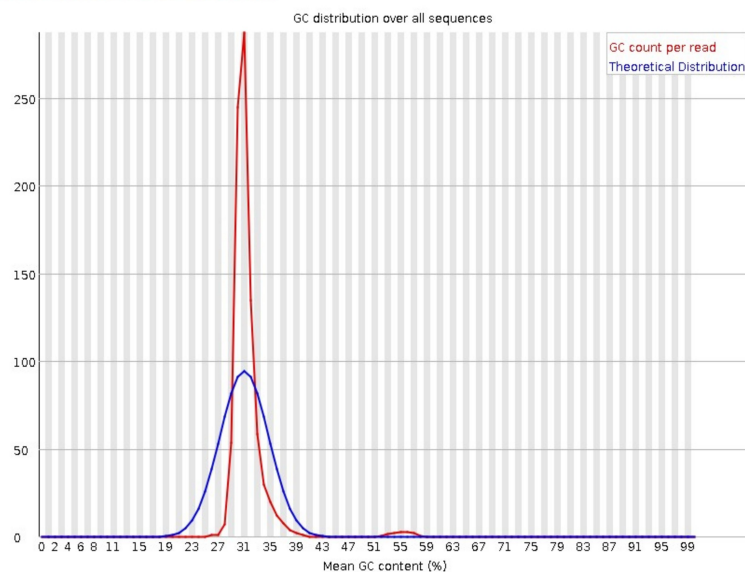

## C.

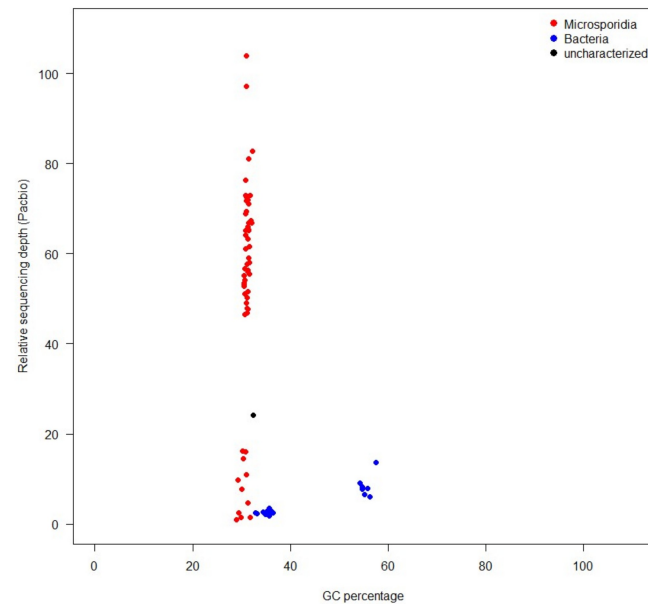

## D.

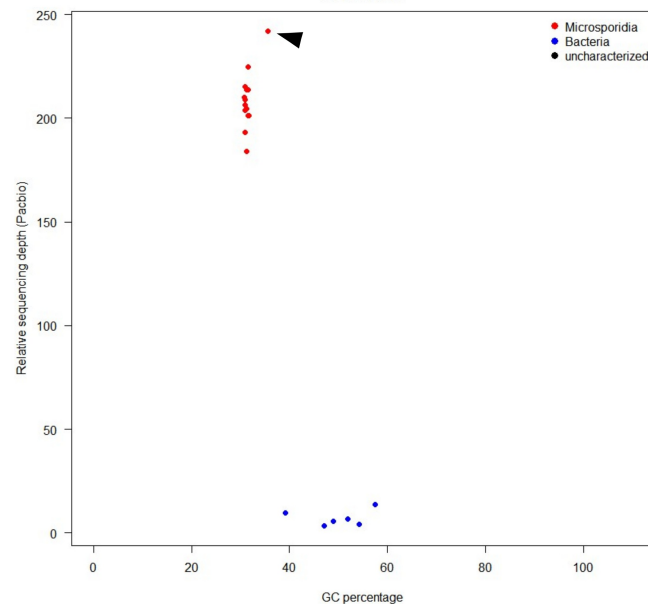

Supplementary Figure 2.

Supplement: Supplementary file 6 — Supplementary Material 6. Fig S2: Overview of reads and assembly of PacBio sequencing dataset before and after host read decontamination, and for haploid and tetraploid assembly conditions. A. FastQC overview of Raw PacBio read distributions of read count against read GC content. Two distinct peaks are visible of approximately 300,000 and 420,000 reads with GC content of 31% and 45%, respectively. B. FastQC overview of PacBio reads after removal of reads aligned to An. gambiae, exhibiting only a single peak of approximately 300,000 reads and 31% GC content. C. SprayNPray output figure of contigs assembled from host decontaminated reads following haploid assembly. The predicted phylogenetic origins of contigs are illustrated through point colour, with uncharacterised and bacterial contigs being clustered with low read depth and high GC content. Contigs for further syntenic alignment were isolated for the elongated cluster of 55 contigs with a GC content of ~31% and >40x sequencing depth. D. SprayNPray output figure of contigs assembled from host decontaminated reads following tetraploid assembly. The predicted phylogenetic origins of contigs are illustrated through point colour. The outlier contig that was excluded from the final genome is indicated with a black arrow. [file 12864_2026_12799_MOESM6_ESM.pdf]
